# Pulmonary Arterial Hypertension Associated with Congenital Heart Disease in Adults over the Age of 40 Years

**DOI:** 10.3390/jcm9124071

**Published:** 2020-12-17

**Authors:** Susanne J. Maurer, Katharina Stöckemann, Claudia Pujol, Jürgen Hörer, Peter Ewert, Oktay Tutarel

**Affiliations:** 1 Department of Congenital Heart Disease and Paediatric Cardiology, German Heart Centre Munich, Technical University of Munich, 80636 Munich, Germany; susanne.maurer@tum.de (S.J.M.); stoeckemann@dhm.mhn.de (K.S.); claupujol@gmail.com (C.P.); ewert@dhm.mhn.de (P.E.); 2 Department of Electrophysiology, German Heart Centre Munich, Technical University of Munich, 80636 Munich, Germany; 3 Department of Congenital and Paediatric Heart Surgery, German Heart Centre Munich, Technical University of Munich, 80636 Munich, Germany; hoerer@dhm.mhn.de; 4 Division of Congenital and Paediatric Heart Surgery, University Hospital Munich, Ludwig-Maximilians Universität, 80337 Munich, Germany; 5 DZHK (German Centre for Cardiovascular Research), Partner Site Munich Heart Alliance, 80992 Munich, Germany

**Keywords:** adult congenital heart disease, pulmonary hypertension, outcome

## Abstract

Background: Pulmonary arterial hypertension associated with adult congenital heart disease (PAH-ACHD) leads to significant mortality at a young age. Risk factors for a negative outcome in older adults are lacking. Methods: PAH-ACHD patients ≥ 40 years of age under active follow-up between January 2005 and December 2018 were included. Demographic data, as well as medical/surgical history, were retrieved from hospital records. The primary end-point was all-cause mortality. Results: In total, 65 patients (67.7% female, mean age 45.19 ± 6.75 years) were included. Out of these, 46 (70.8%) had a shunt lesion, 12 (18.5%) had PAH associated with complex congenital heart defects, and 7 (10.8%) had segmental pulmonary hypertension due to major aorto-pulmonary collaterals. Down syndrome was present in 13 patients (20.0%). During a median follow-up of 4.2 years (IQR 1.2–7.5), 16 patients (24.6%) died. On univariate analysis, NT-proBNP (log), creatinine, and a previous history of ventricular arrhythmias were predictors of all-cause mortality. Upon multivariate analysis, NT-proBNP (log) (HR: 4.1, 95% CI: 1.2–14.4, *p* = 0.029) and creatinine (HR: 16.3, 95% CI: 2.2–118.7, *p* = 0.006) remained as independent predictors of all-cause mortality. Conclusions: PAH-ACHD patients over the age of 40 years are burdened with significant mortality, of which NT-proBNP and creatinine are independent predictors.

## 1. Introduction

The life expectancy for those born with congenital heart disease (CHD) has greatly improved over the last few decades [1]. Consequently, the population of adults with congenital heart disease (ACHD) is growing and patients are increasingly reaching older ages [2]. This is not only true for patients with simple defects, but also for those with complex defects [3]. Accordingly, the median age of ACHD patients has increased to 40 years [1]. ACHD patients with pulmonary arterial hypertension (PAH-ACHD) are at the extreme end of this spectrum with one of the highest mortality rates and significant morbidity and mortality [4,5,6]. Nonetheless, even in these patients a shift towards mortality at an older age, as well as a median survival of 52 years, has been reported [5]. While risk factors for a worse prognosis are well described in younger cohorts [6], data regarding older PAH-ACHD patients are scarce. Therefore, the aim of this study is to examine the clinical course of patients with PAH-ACHD over the age of 40 years, and to identify possible risk factors for a worse prognosis.

## 2. Materials and Methods

This study complies with the Declaration of Helsinki, and the ethics committee of the Medical Faculty of the Technical University of Munich has approved the research protocol (Protocol number 122/19 S). The requirement for informed consent was waived by the ethics committee due to the retrospective nature of the study.

This retrospective, single-center study included all patients with a diagnosis of PAH-ACHD under follow-up at the German Heart Centre Munich who were ≥40 years of age at any point between January 2005 and December 2018. The time-point of inclusion was either the date of the 40th birthday or, if the patient was already 40 years old before the year 2005, the first visit after the 1 January 2005.

PAH-ACHD was established and confirmed by echocardiography, cardiac MRI, and/or cardiac catheterization. The underlying CHD was classified as follows: (1) Shunt lesions—atrial septal defects (ASD), ventricular septal defects (VSD), atrioventricular septal defects (AVSD), persistent ductus arteriosus, aortopulmonary window; (2) Complex—PAH associated with complex CHD, e.g., transposition of the great arteries after atrial switch surgery with remaining VSD or univentricular hearts; (3) Segmental—PAH due to major aorto-pulmonary collateral arteries. The latter was diagnosed according to recently proposed criteria [7].

The primary end-point was all-cause mortality. Demographic data and information on medical/surgical history were retrieved from hospital records. Symptomatic status was assessed according to the New York Heart Association classification (NYHA). Based on the results of routine transthoracic echocardiograms, left and right ventricular systolic function was graded semi-quantitatively as normal, or mildly, moderately or severely impaired, as described previously [8]. Arrhythmias encompassed any type of supraventricular or ventricular arrhythmia requiring treatment.

The data associated with the paper are not publicly available.

Statistical analyses were performed using SPSS version 25 (IBM Corp., Armonk, NY, USA) and MedCalc version 19.0.3.0 (MedCalc Software, Mariakerke, Belgium). Continuous variables are presented as mean ± standard deviation or median (interquartile range), whereas categorical variables are presented as number (percentage). Comparison between groups was performed using the Mann–Whitney U test or Student’s *t*-test for continuous and Chi-square test for categorical variables. Univariate Cox proportional hazards analysis was used to assess the association between variables and the primary endpoint. Significant variables (*p* < 0.05) were subsequently included in a multivariate Cox proportional hazards analysis model in a stepwise fashion.

Cut-off values for significant variables from the multivariate Cox proportional hazards analysis were derived using the Youden index with a receiver operating characteristics (ROC) curve. Kaplan–Meier curves and log-rank tests were used to compare survival (all-cause mortality) between patients with values below and above the cut-off of significant variables.

All tests were performed two-sided, and for all analyses a *p*-value < 0.05 was considered statistically significant.

## 3. Results

Overall, 65 patients (female 67.7%, mean age 45.19 ± 6.75 years) were included. Out of these, 46 patients (70.8%) had a shunt lesion, 12 (18.5%) a complex CHD, and 7 (10.8%) segmental PAH-ACHD. Down syndrome was present in 13 patients (20.0%). A medication with advanced PAH therapies was present in 26 patients (40.0%); out of these, 18 received a monotherapy and eight a combination therapy of two drugs. More detailed information regarding the baseline characteristics is provided in Table 1.

During a median follow-up of 4.2 years (IQR 1.2–7.5), thromboembolic events occurred in nine patients (13.8%), hemoptysis in 2 (3.1%), atrial arrhythmias in 15 (23.1%), and ventricular arrhythmias in 6 (9.2%). An electrophysiological study was performed in four patients (6.2%) and a cardioversion in 12 (18.5%). Surgery for electrophysiological devices was performed in 12 patients (18.5%), including an implantable cardioverter defibrillator (ICD) implantation in 4, event recorder implantation in 5, and a box change in 3 (2 pacemaker, 1 ICD). Hospitalizations occurred in 47 patients (72.3%); out of these, 21 were unplanned/emergency admissions. Causes for the latter were cardiac in 18, infections in 2, and hemoptysis in 1.

During follow-up, 16 patients (24.6%) died. The cause of death was cardiac in three patients, respiratory failure in one, cerebrovascular accident in one, and unknown in eleven patients. Upon univariate analysis, NT-proBNP (log) (HR: 3.5, 95% CI: 1.1–11.6, *p* = 0.037), creatinine (HR: 12.8, 95% CI: 2.1–79.3, *p* = 0.006), and a previous history of ventricular arrhythmias (HR: 5.1, 95% CI: 1.0–25.9, *p* = 0.0497) were predictors of all-cause mortality (Table 2). Upon multivariate analysis, NT-proBNP (log) (HR: 4.1, 95% CI: 1.2–14.4, *p* = 0.029) and creatinine (HR: 16.3, 95% CI: 2.2–118.7, *p* = 0.006) remained as independent predictors.

**Figure 1:**
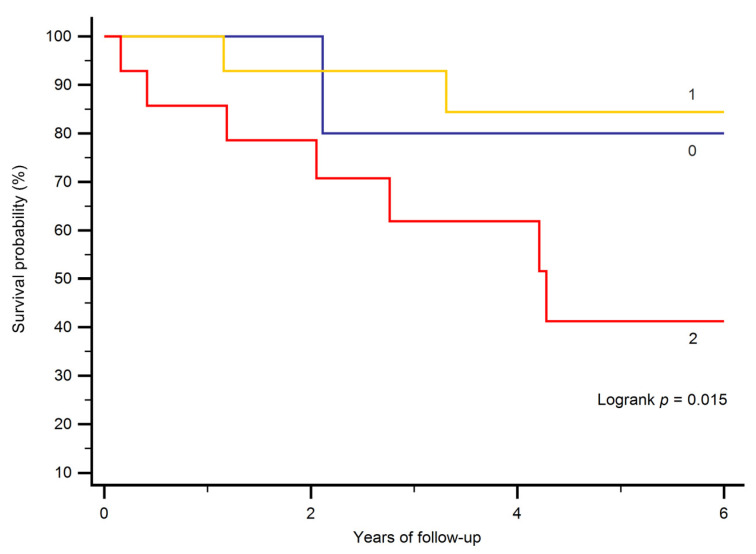
Kaplan–Meier curves stratifying patients according to the presence of laboratory results above or below the cut-off value for NT-proBNP and creatinine. 0 indicates that neither was above the cut-off value, 1 indicates that one (either NT-proBNP or creatinine) was above its respective cut-off value, and 2 indicates that both were above the respective cut-off values.

The cut-off value for NT-proBNP was 1250 ng/L, and for creatinine 0.73 mg/dL. In patients in whom both values were above the cut-off value, survival was diminished (*p* = 0.015; Figure 1).

## 4. Discussion

Patients with PAH-ACHD over the age of 40 years are burdened with significant morbidity and mortality. During a median follow-up of four years, approximately one-third of the patients required unplanned/emergency hospital admissions, and a quarter of the patients died. The independent predictors of mortality were NT-proBNP and creatinine.

Kempny et al. analyzed predictors of death in Eisenmenger patients in a large multicenter study of 1098 patients [6]. The median age of their study cohort was 34 years, and over a median follow-up of 3.1 years, 278 patients (25.3%) died. This is similar to the mortality of 24.6% over 4.2 years observed in our older cohort, pointing to a significant risk of death even in those patients with PAH-ACHD who “made it” to their 40th birthday. Considering predictors of death, brain natriuretic peptide (BNP) was a predictor of mortality in the univariable analysis in the multicenter study, but did not remain in the multivariable model [6]. Interestingly, in accordance with our results, BNP predicted survival independently of renal function, Down syndrome, or 6 min walk test distance in a large single-center study [9]. Additional studies are necessary to define the role of BNP/NT-proBNP in these patients. Furthermore, creatinine—an independent predictor of mortality in our cohort—was not included in the study by Kempny and colleagues [6]. However, the prognostic value of renal dysfunction was proven in 1102 ACHD patients with a variety of underlying CHD [10]. Mortality was three-fold higher than normal in patients who had moderate or severely reduced renal dysfunction according to a creatinine-based glomerular filtration rate (GFR) equation [10]. Interestingly, a decrease in GFR was more common among patients with Eisenmenger physiology, of whom 72% had reduced GFR, and in 18% this was moderately or severely reduced [10]. Therefore, renal dysfunction as an important predictor of mortality needs to be taken into account. Regarding the risk assessment of these patients, the combination of elevated creatinine and NT-proBNP levels indicated a high-risk group in our cohort. Patients in whom both values were above the cut-off value had diminished survival.

Treatment with advanced PAH therapies was not predictive of mortality in our study and it was also not an independent predictor in the multicenter study [6]. Regarding our study, this is probably because the number of patients included is too small to prove a benefit of drug treatment. Furthermore, while advanced PAH therapies have been shown to benefit patients in multiple ways, for example improving 6 min walk distance and functional class [11], survival benefit in a prospective study has not been shown yet. At least a signal of a survival benefit can be derived from retrospective studies in larger cohorts [12,13]. Interestingly, only 40% of our patients were on advanced PAH therapies at baseline. This is probably due to a few reasons. First, the study period started in 2005, but the BREATHE-5 trial that proved the benefit of a therapy with bosentan in patients with Eisenmenger syndrome was not published until 2006 [11]. Additionally, the uptake of these therapies in the cohort of PAH-ACHD patients was slow, such that even 10 years later an analysis of the data from the German National Register for congenital heart defects found that more than 40% of patients did not receive advanced PAH therapies [13].

Hospitalizations were necessary in 72% of patients in our cohort. Out of these, almost a half were unplanned/emergency admissions, mainly due to cardiac reasons. Notably, arrhythmias and their treatment were a significant cause for health care utilization. A progressive increase in admissions of ACHD patients in general in the last two decades has been reported [14]. The number of admissions for PAH-ACHD more than doubled during a ten-year span in the United States [14]. Our results indicate an already significant use of healthcare resources in our cohort, which probably will increase even further as these patients are getting older, considering that the elderly ACHD population has an even higher rate of health care utilization compared to younger ACHD patients [3]. The number of unplanned hospitalizations in our cohort is in line with previously reported figures for complex CHD, which range between 17% for patients with tetralogy of Fallot and 52% for patients with single ventricle physiology [15]. A leading cause of hospitalizations in patients with complex CHD is arrhythmias [15]. Furthermore, a new arrhythmia episode was also common in PAH-ACHD patients in a recent study, and was associated with an adverse long-term outcome [16]. In our cohort, a previous history of ventricular arrhythmias was a predictor of all-cause mortality in the univariate analysis, but not in the multivariate analysis. This may be due to the small number of patients in our cohort. Considering that arrhythmias in PAH-ACHD were a strong predictor of mortality even when managed in a specialist center [16], new approaches regarding their prevention and treatment seem to be necessary.

A limitation of our study is the small number of patients included from a heterogeneous population. Another limitation is the retrospective nature of the study, with all its obvious drawbacks. In some patients, variables such as NT-proBNP were missing. Furthermore, the 6 min walk test as an objective measure of exercise capacity was not regularly performed. Additionally, the cause of death was not available in the majority of deceased patients.

## 5. Conclusions

In conclusion, in patients with PAH-ACHD over the age of 40 years, a substantial number of unplanned/emergency hospital admissions as well as significant mortality were observed. Close surveillance, including regular blood analysis, is necessary to identify patients at risk.

## Figures and Tables

**Table 1:** Baseline characteristics.

	All	Dead	Alive	*p*
*n*	65	16	49	
Age (years)	45.2 ± 6.8	43.9 ± 5.4	45.6 ± 7.1	0.29
Female, *n* (%)	44 (67.7)	11 (68.8)	33 (67.3)	0.92
Congenital heart defect, *n* (%)				0.11
Shunt	46 (70.8)	8 (50.0)	38 (77.6)	
Complex	12 (18.5)	5 (31.3)	7 (14.3)	
Segmental	7 (10.8)	3 (18.8)	4 (8.2)	
Down syndrome, *n* (%)	13 (20.0)	4 (25.0)	9 (18.4)	0.57
NT-proBNP (ng/L)	1838 ± 2088	2702 ± 2810	1464 ± 1602	0.06
Creatinine (mg/dL)	0.91 ± 0.26	1.02 ± 0.34	0.87 ± 0.22	0.21
CRP (mg/L)	13.94 ± 34.60	9.07 ± 9.36	15.99 ± 40.92	0.64
History of				
Atrial arrhythmias	11 (16.9)	4 (25.0)	7 (14.3)	0.32
Ventricular arrhythmias	5 (7.7)	2 (12.5)	3 (6.1)	0.41
Hemoptysis	10 (15.4)	1 (6.3)	9 (18.4)	0.24
Thromboembolic events	18 (27.7)	5 (31.3)	13 (26.5)	0.71
Advanced PAH therapies	26 (40.0)	4 (25.0)	22 (44.9)	0.16
NYHA class, *n* (%)				0.13
II	29 (59.2)	8 (80.0)	21 (53.8)	
III–IV	20 (40.8)	2 (20.0)	18 (46.2)	
LV function, *n* (%)				0.48
normal	44 (81.5)	10 (76.9)	34 (82.9)	
mild-moderate	8 (14.8)	3 (23.1)	5 (12.2)	
severe	2 (3.7)	0 (0)	2 (4.9)	
RV function, *n* (%)				0.09
normal	32 (64.0)	5 (41.7)	27 (71.1)	
mild–moderate	15 (30.0)	5 (41.7)	10 (26.3)	
severe	3 (6.0)	2 (16.7)	1 (2.6)	

CRP: C-reactive protein; LV: left ventricular; NYHA: New York Heart Association; NT-proBNP: N-terminal pro–B-type natriuretic peptide; PAH: pulmonary arterial hypertension; RV: right ventricular; *p* value is for comparison between patients who died and those that are alive (Mann–Whitney U test was used for continuous and Chi-square test for categorical variables).

**Table 2:** Univariate and multivariate analysis of predictors for all-cause mortality.

Variable	Univariate		Multivariate	
	HR (95% CI)	*p*	HR (95% CI)	*p*
Age	0.99 (0.90–1.08)	0.80		
Atrial arrhythmias	1.26 (0.40–3.96)	0.69		
CRP	0.99 (0.96–1.03)	0.76		
Creatinine	12.76 (2.05–79.32)	0.0063	16.28 (2.23–118.69)	0.0059
Complex vs. Shunt	1.39 (0.45–4.31)	0.57		
Segmental vs. Shunt	2.04 (0.54–7.75)	0.30		
Male	1.06 (0.37–3.05)	0.92		
Reduced LV function	1.20 (0.33–4.38)	0.78		
NYHA functional class	0.41 (0.10–1.69)	0.22		
Oxygen saturation	0.96 (0.88–1.05)	0.37		
Reduced RV function	3.09 (0.97–9.88)	0.06		
Liver disease	3.08 (0.85–11.13)	0.09		
Down syndrome	1.65 (0.52–5.21)	0.40		
Systemic RV vs. systemic LV	1.80 (0.51–6.33)	0.36		
NT-proBNP (log)	3.54 (1.08–11.64)	0.037	4.08 (1.16–14.41)	0.0289
Ventricular arrhythmias	5.09 (1.00–25.86)	0.0497		

HR indicates hazard ratio; NYHA indicates New York Heart Association; LV indicates left ventricular and RV right ventricular.
